# Impact of left subclavian artery management on surgical efficiency and perioperative cerebral infarction in type A aortic dissection under deep hypothermic circulatory arrest: a single-center retrospective cohort study

**DOI:** 10.3389/fcvm.2026.1855005

**Published:** 2026-09-09

**Authors:** Yun Lu, Haisu Xu, Yang Zhang, Jun Wei, Fangyuan Chen

**Affiliations:** 1 Department of Cardiology, The Second Affiliated Hospital of Xuzhou Medical University, Xuzhou, China; 2 Department of Cardiac Surgery, Xuzhou Central Hospital, Southeast University, Xuzhou, China; 3 Department of Cardiothoracic Surgery, Affiliated Hospital of Xuzhou Medical University, Xuzhou, China

**Keywords:** aortic dissection, cerebral infarction, deep hypothermic circulatory arrest (DHCA), hemiarch replacement, left subclavian artery (LSA)

## Abstract

**Background:**

Acute type A aortic dissection is a catastrophic cardiovascular emergency requiring urgent surgery. Hemiarch replacement under deep hypothermic circulatory arrest (DHCA) is a standard approach. This study compared the outcomes of two supra-aortic vessel management strategies during DHCA.

**Methods:**

This retrospective cohort study analyzed 109 patients undergoing hemiarch replacement between May 2024 and January 2026. Patients were divided into two groups: the left subclavian artery (LSA) Occlusion Group (*n* = 53), in which all three arch vessels (innominate, left common carotid, and left subclavian arteries) were dissected and clamped, and the LSA Non-Occlusion Group (*n* = 56), in which the LSA was left untouched. Continuous and categorical variables were compared using the Mann–Whitney U test and Chi-square/Fisher's exact test, respectively. Multivariable logistic regression was performed to identify independent risk factors for perioperative cerebral infarction.

**Results:**

Preoperative baseline characteristics were well-balanced between groups (all *p* > 0.05). The LSA Occlusion Group had significantly longer total surgical duration (320 vs. 273 min, *p* < 0.001), aortic cross-clamp time (126 vs. 104.5 min, *p* = 0.016), and DHCA time (26 vs. 22 min, *p* < 0.001). The incidence of perioperative cerebral infarction was significantly higher in the LSA Occlusion Group (22.64% vs. 7.14%, *p* = 0.044). Multivariable analysis confirmed that LSA occlusion was an independent risk factor for cerebral infarction (OR=4.36, 95% CI 1.24–15.30, *p* = 0.020). The 30-day mortality rate was numerically higher in the LSA Occlusion Group (20.75% vs. 10.71%) but did not reach statistical significance (*p* = 0.238). No significant differences were found in other major complications or recovery metrics.

**Conclusion:**

In this cohort, the strategy of clamping the left subclavian artery during hemiarch replacement was associated with prolonged critical intraoperative times and a significantly increased risk of perioperative cerebral infarction. LSA occlusion was identified as an independent risk factor for stroke. These findings suggest that omitting LSA manipulation may be a safer and more efficient approach, but the results require validation in larger, prospective studies.

## 1 Introduction

Acute type A aortic dissection (ATAAD) represents one of the most catastrophic cardiovascular emergencies, characterized by a fulminant clinical course and an exceedingly high mortality rate if left untreated. The pathophysiology involves a tear in the intimal layer of the ascending aorta, allowing blood to dissect through the media, which can rapidly lead to aortic rupture, cardiac tamponade, or severe malperfusion of vital organs. Epidemiological data indicate that without immediate surgical intervention, the mortality rate for ATAAD increases by approximately 1% to 2% per hour during the initial 48 h following symptom onset, underscoring the critical nature of timely diagnosis and management. While advancements in diagnostic imaging and perioperative care have improved survival rates over recent decades, ATAAD continues to impose a substantial burden on healthcare systems globally, necessitating complex surgical procedures, prolonged intensive care unit stays, and extensive long-term rehabilitation. Consequently, surgical repair remains the only definitive therapeutic strategy capable of preventing fatal outcomes, with the primary goals being the resection of the primary entry tear, reconstruction of the aortic root and ascending aorta, and restoration of true lumen flow to branch vessels.

Despite the established necessity of surgical intervention, significant controversy persists regarding the optimal extent of aortic arch reconstruction during the initial repair of ATAAD. The traditional approach often involves hemiarch replacement under deep hypothermic circulatory arrest (DHCA), which balances the urgency of life-saving surgery with the risks associated with prolonged operative times and complex arch manipulation. However, the management of supra-aortic branch vessels, particularly the left subclavian artery (LSA), remains a subject of intense debate among cardiac surgeons. Some proponents advocate for aggressive total arch replacement or extended hemiarch techniques that include the reimplantation or bypass of all three arch branches to ensure complete false lumen thrombosis and reduce the need for future distal reoperations (1). Conversely, other studies suggest that a more conservative hemiarch approach, potentially sparing the LSA from direct manipulation, may yield comparable survival outcomes while minimizing the duration of cerebral ischemia and systemic inflammatory response (2). The decision to clamp or preserve the LSA is further complicated by the potential risk of spinal cord ischemia, left upper extremity malperfusion, and the development of subclavian steal syndrome, especially in patients with prior coronary artery bypass grafting using the left internal mammary artery (3).

Current literature provides conflicting evidence regarding the safety and efficacy of different strategies for managing the LSA during arch reconstruction. On one hand, coverage or occlusion of the LSA during thoracic endovascular aortic repair (TEVAR) or open surgery has been associated with increased risks of posterior circulation stroke and upper limb ischemia, particularly in trauma populations or those with incomplete Circle of Willis anatomy (4). Furthermore, the presence of LSA stenosis or occlusion can precipitate coronary-subclavian steal syndrome in patients with LIMA grafts, leading to acute myocardial infarction and hemodynamic instability (5). On the other hand, recent comparative analyses indicate that extending the repair to include zone 2 or total arch replacement does not necessarily confer a survival advantage over hemiarch replacement in the acute setting, yet it significantly prolongs cardiopulmonary bypass and circulatory arrest times (6). Moreover, aggressive arch replacement has been linked to higher rates of reoperation for bleeding and increased transfusion requirements, which are independent predictors of poor prognosis (7). Despite these insights, there remains a paucity of high-quality data specifically evaluating the impact of selectively omitting LSA clamping during standard hemiarch replacement on short-term neurological outcomes and overall procedural efficiency.

The existing research gap lies in the lack of granular analysis comparing the “three-vessel clamping” strategy versus a “two-vessel clamping” strategy (sparing the LSA) specifically within the context of standardized hemiarch replacement for ATAAD. While previous studies have broadly compared hemiarch versus total arch replacement, few have isolated the specific contribution of LSA management to the duration of deep hypothermic circulatory arrest and its subsequent effect on organ protection (8). Additionally, the relationship between the duration of circulatory arrest and the incidence of specific complications, such as perioperative cerebral infarction and renal failure, requires further elucidation in the context of simplified surgical workflows (9). It is hypothesized that omitting the dissection and clamping of the LSA may streamline the surgical procedure, thereby reducing the critical window of global ischemia without compromising cerebral perfusion or increasing the risk of distal aortic events. Addressing this uncertainty is crucial for refining surgical protocols and optimizing resource utilization in high-volume aortic centers.

To address these unresolved questions, the present study employs a retrospective cohort design to systematically evaluate the clinical outcomes of two distinct supra-aortic vessel management strategies in patients undergoing hemiarch replacement for ATAAD. By leveraging real-world clinical data from a specialized cardiac surgery center, this investigation uses multivariable logistic regression models to control for potential confounding factors such as patient demographics, comorbidities, and anatomical variations. This methodological approach, while mitigating some limitations of single-center retrospective analyses, allows for a precise assessment of the association between surgical technique and perioperative metrics (10). Specifically, the use of generalized linear models enables the identification of independent risk factors for adverse outcomes, providing a quantitative framework to distinguish the effects of surgical duration from those of intrinsic patient pathology. This analytical strategy ensures that the observed differences in outcomes can be attributed with greater confidence to the variation in LSA handling rather than baseline disparities.

The primary objectives of this research are threefold: first, to compare the operative efficiency of the two strategies by analyzing key time-dependent variables, including total surgical duration, aortic cross-clamp time, and, most critically, the duration of deep hypothermic circulatory arrest; second, to evaluate the safety profile of sparing the LSA by assessing the incidence of major postoperative complications, with a specific focus on neurological deficits, renal dysfunction, and in-hospital mortality; and third, to identify independent predictors of perioperative cerebral infarction via multivariable logistic regression, which may inform future risk stratification models. By delineating the trade-offs between procedural simplification and potential ischemic risks, this study aims to provide evidence to guide surgical decision-making in ATAAD repair. Ultimately, the findings seek to contribute to the ongoing discourse on the extent of arch replacement, offering insights that could lead to reduced operative times, enhanced organ protection, and improved overall survival for patients facing this lethal condition (11). Through this comprehensive evaluation, we aspire to clarify the role of the left subclavian artery in modern aortic surgery and support the development of more refined, patient-specific treatment algorithms.

## 2 Materials and methods

### 2.1 Study design and patient population

This study was conducted at the Department of Cardiothoracic Surgery, Xuzhou Medical University Affiliated Hospital. We retrospectively enrolled patients diagnosed with Type A aortic dissection who underwent surgical repair between May 2024 and January 2026. All included patients underwent hemi-arch replacement under deep hypothermic circulatory arrest (DHCA). This study was approved by the hospital's Institutional Review Board. Informed consent was waived due to the retrospective nature of the study.

### 2.2 Study selection criteria

#### 2.2.1 Inclusion criteria (must meet all)

- Age: >18 years.
- Diagnosis: Confirmed by imaging (CTA or MRA) as Stanford type A aortic dissection, with the dissection limited solely to the ascending aorta and without extension into the aortic arch or descending aorta.
- Surgical Procedure: Undergoing ascending aortic replacement with hemiarch replacement.

#### 2.2.2 Exclusion criteria (excluded if any are met)

Dissection Extent & Surgical Indication:

- Dissection involving the aortic arch, descending thoracic aorta, or abdominal aorta, necessitating total aortic arch replacement with frozen elephant trunk procedure.
- Surgery not involving hemiarch replacement or not requiring deep hypothermic circulatory arrest (e.g., isolated ascending aortic replacement, Bentall procedure, David procedure).
- Having undergone or scheduled for total aortic arch replacement with frozen elephant trunk procedure.
- Surgical History: Previous history of any aortic surgery (e.g., thoracic endovascular aortic repair, open aortic surgery).
- Presence of malignant tumors, severe hepatic or renal dysfunction, coagulation disorders, or other diseases that may compromise surgical evaluation.
- Active infection or uncontrolled systemic inflammatory state.
- Other Factors: (Pregnancy or lactation; Incomplete clinical data or anticipated difficulty with follow-up)

These criteria are designed to select a homogeneous cohort of patients with type A dissection confined to the ascending aorta, treated specifically with ascending aorta and hemiarch replacement, for focused clinical outcome analysis.

### 2.3 Grouping and surgical techniques

Patients were stratified into two groups based on the management of the aortic arch branch vessels during deep hypothermic circulatroy arrest (DHCA):

1. LSA Occlusion Group: Three-Vessel Occlusion Group: In this group, all three branch vessels of the aortic arch were dissected and individually clamped during DHCA, followed by ascending aorta replacement.
2. LSA Non-Occlusion Group: Two-Vessel Occlusion Group: In this group, only two branch vessels—the innominate artery and the left common carotid artery—were dissected and clamped. The left subclavian artery (LSA) was neither dissected nor clamped.

The decision to occlude or spare the left subclavian artery (LSA) was determined preoperatively by the attending surgeon based on the anatomical characteristics of the dissection as visualized on computed tomography angiography (CTA). Specifically, the LSA was occluded (LSA Occlusion Group) if the dissection flap was observed to extend into or was deemeLd to compromise the orifice of the LSA, or if clamping was considered necessary to ensure a bloodless surgical field for the distal anastomosis. Conversely, the LSA was spared (LSA Non-Occlusion Group) when the dissection was limited to the ascending aorta without any evidence of involvement of the LSA ostium, and the surgeon judged that the hemiarch anastomosis could be performed safely without clamping the LSA.

#### 2.3.1 Operative technique

**Figure 1:**
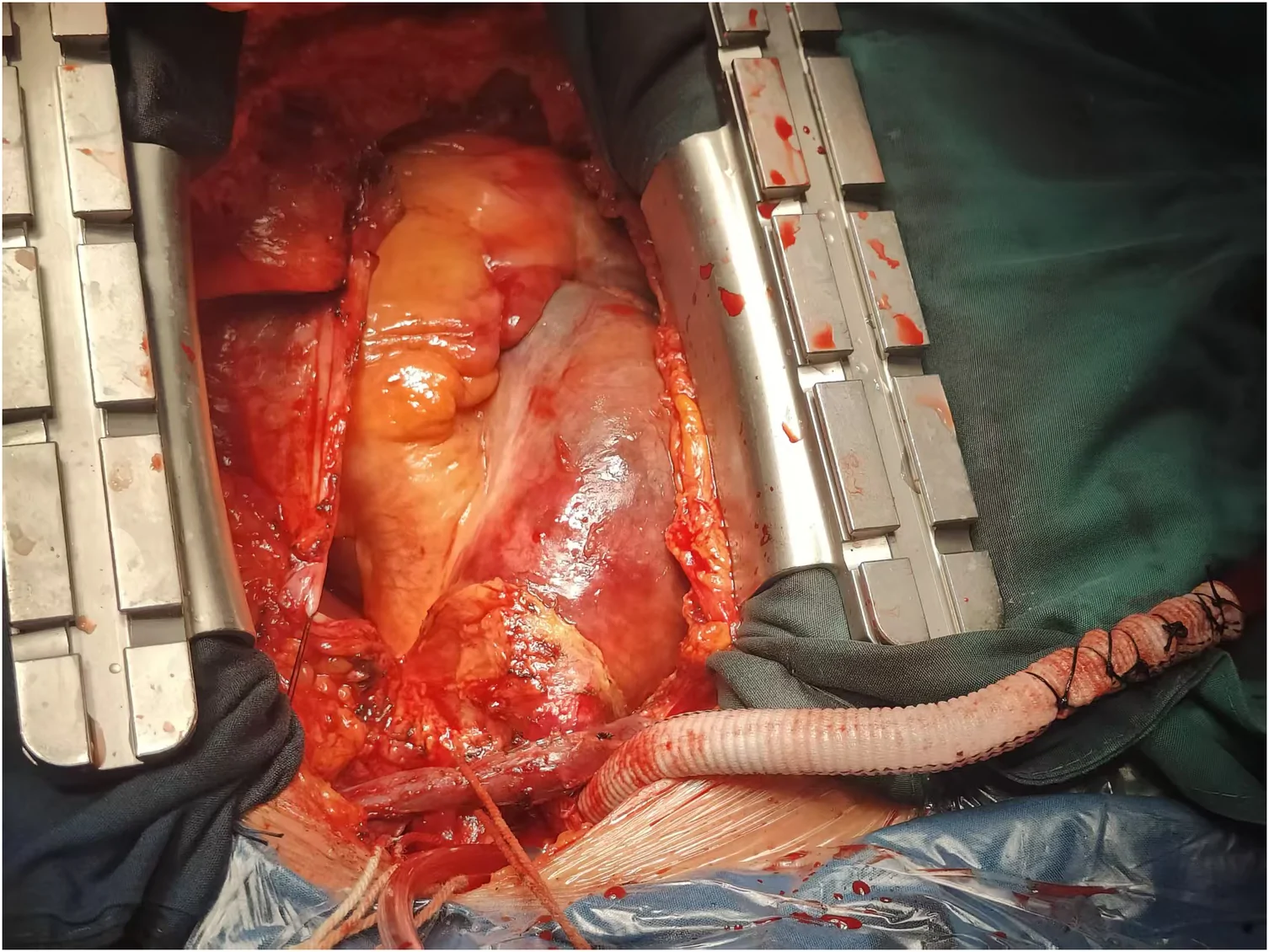
End-to-side anastomosis between the innominate artery and a 10-mm Dacron graft, which is used for systemic perfusion and unilateral cerebral perfusion during deep hypothermic circulatory arrest.

All procedures were performed via a standard median sternotomy. Arterial cannulation was routinely achieved via the innominate artery using an 10-mm Dacron graft Perform an anastomosis, with venous drainage via the right atrium (Figure 1). After the initiation of cardiopulmonary bypass, the patient was cooled to a target nasopharyngeal temperature of 22–24 °C. Myocardial protection was achieved with Del Nido cardioplegia delivered every 90 min.

During deep hypothermic circulatory arrest (DHCA), selective antegrade cerebral perfusion (SACP) was established via the the innominate artery using an 10-mm Dacron graft Perform an anastomosis, with a flow rate of 5–8 mL/kg/min, maintaining a right radial artery pressure of 40–60 mmHg. In the LSA Occlusion Group, all three arch vessels (innominate artery, left common carotid artery, and left subclavian artery) were dissected and clamped during DHCA. In the LSA Non-Occlusion Group, only the innominate and left common carotid arteries were clamped; the LSA was left untouched. The open distal anastomosis of the hemiarch was performed during a single period of DHCA. After completion of the distal anastomosis, CPB was resumed via the graft side branch, and the patient was rewarmed to a bladder temperature of 35 °C.

Management of Back-Bleeding from the LSA in the Non-Occlusion Group: In the LSA Non-Occlusion Group, where the LSA was not clamped, back-bleeding from the LSA into the operative field during DHCA was effectively managed by a combination of techniques. First, the innominate and left common carotid arteries were clamped immediately after the initiation of DHCA, creating a low-flow, low-pressure environment in the arch. Second, a small, soft-jawed vascular clamp or a silicone elastomer loop was placed around the origin of the LSA from within the opened arch, but was not fully occluded; instead, it was used to gently tamponade the LSA orifice. This simple maneuver, easily performed through the arch incision, typically reduced back-bleeding to a negligible level without the need for a separate dissection or complete clamping of the vessel. Third, the continuous low-flow suction from the left-sided heart vent and the cardiotomy suction within the open arch effectively cleared any residual blood, ensuring a clear field for the distal anastomosis. This approach avoids the potential embolic and hemodynamic risks associated with a full LSA clamping, as described in the LSA Occlusion Group.

### 2.4 Definitions of clinical outcomes

The following standardized definitions were used for the primary and secondary outcomes:

1. Perioperative cerebral infarction was defined as a new neurological deficit of any duration confirmed by cranial computed tomography (CT) or magnetic resonance imaging (MRI) during the hospitalization. Brain imaging was performed when clinically indicated by new neurological symptoms or signs; routine postoperative imaging was not performed.
2. Acute kidney injury (AKI) was defined according to the kidney disease: improving global outcomes (KDIGO) criteria (12): an increase in serum creatinine by ≥0.3 mg/dL (≥26.5 μmol/L) within 48 h, or an increase in serum creatinine to ≥1.5 times baseline within 7 days.
3. Perioperative myocardial infarction was defined according to the fourth universal definition of myocardial infarction: a rise and/or fall of cardiac troponin values with at least one value above the 99th percentile upper reference limit, and at least one of the following: symptoms of myocardial ischemia; new ischemic electrocardiographic changes; development of pathological Q waves; or imaging evidence of new loss of viable myocardium.
4. 30-day mortality was defined as death from any cause within 30 days of the index surgery.

### 2.5 Statistical analysis

Baseline characteristics, intraoperative parameters, and postoperative outcomes were compared between the two groups. Continuous variables were analyzed using the Wilcoxon rank-sum test. Categorical variables, including demographic data, comorbidities, anatomical involvement, and complication rates, were compared using the Chi-square test or Fisher's exact test where appropriate. Furthermore, a generalized linear model with logistic regression was employed to identify independent risk factors associated with clinical outcomes. A *p*-value of less than 0.05 was considered statistically significant.

## 3 Result

A total of 109 patients with acute Type A aortic dissection who underwent hemiarch replacement under deep hypothermic circulatory arrest (DHCA) were included in this retrospective study. Based on the surgical strategy for the supra-aortic branches during DHCA, patients were divided into two groups: the LSA Non-Occlusion Group (*n* = 56), where only two vessels (innominate and left common carotid arteries) were dissected and occluded (sparing the LSA), and the LSA Occlusion Group (*n* = 53), where all three branch vessels were occluded.

### 3.1 Baseline characteristics

The preoperative demographic and clinical characteristics of the two groups are summarized in Table 1. There were no statistically significant differences between the two groups in terms of age, BMI, heart rate, preoperative cardiac biomarkers (NT-proBNP and high-sensitivity cardiac troponin), sex distribution, smoking history, hypertension, type 2 diabetes mellitus, history of cerebral infarction, atrial fibrillation, cardiogenic shock, cardiac tamponade, or the extent of aortic dissection involving the sinus of Valsalva, innominate artery, or coronary arteries (all *p* > 0.05). This indicates that the two groups were well-balanced at baseline.

**Table 1:** Baseline characteristics.

Variable Names	Level	LSA Non-Occlusion Group	LSA Occlusion Group	*p*
n		56	53	
Age		61.5 (47.75–69.25)	57 (42–69)	0.178
BMI		25.618 (21.905–26.414)	25.391 (24.167–26.122)	0.515
Roomheart		77.5 (70.75–86.75)	80 (76–88)	0.215
Preoperative NT-proBNP level		490.5 (112.5–1,572.75)	355 (104–870)	0.471
Preoperative high-sensitivity cardiac troponin		21.6 (7.075–59.775)	17.7 (7.1–44.2)	0.594
Sex (%)	M	44 (78.57)	44 (83.02)	0.556
	F	12 (21.43)	9 (16.98)	
Smoke (%)	No	48 (85.71)	48 (90.57)	0.435
	Yes	8 (14.29)	5 (9.43)	
Hypertension (%)	No	16 (28.57)	15 (28.30)	0.354
	Yes	40 (71.43)	38 (71.70)	
T2DM (%)	No	54 (96.43)	51 (96.23)	0.955
	Yes	2 (3.57)	2 (3.77)	
Prior cerebral infarction (%)	No	53 (94.64)	50 (94.34)	0.945
	Yes	3 (5.36)	3 (5.66)	
Atrial fibrillation (%)	No	54 (96.43)	53 (100.00)	0.165
	Yes	2 (3.57)	0 (0.00)	
Cardiogenic shock (%)	No	45 (80.36)	45 (84.91)	0.532
	Yes	11 (19.64)	8 (15.09)	
Cardiac tamponade (%)	No	36 (64.29)	38 (71.70)	0.407
	Yes	20 (35.71)	15 (28.30)	
Sinus involvement (%)	No	30 (53.57)	24 (45.28)	0.387
	Yes	26 (46.43)	29 (54.72)	
Innominate artery involvement (%)	No	54 (96.43)	51 (96.23)	0.955
	Yes	2 (3.57)	2 (3.77)	
Coronary artery involvement (%)	No	51 (91.07)	52 (98.11)	0.140
	Yes	5 (8.93)	1 (1.89)	

### 3.2 Intraoperative and early postoperative data

Key intraoperative and early postoperative parameters are presented in Table 2. Continuous variables, which did not follow a normal distribution, were compared using the Mann–Whitney U test. Significant differences were observed in several time-related metrics. The LSA Occlusion Group had a significantly longer total surgical duration (320 [295−350] vs. 273 [248.75–295] min, *p* < 0.001), aortic cross-clamp time (126 [101–130] vs. 104.5 [88.5–122] min, *p* = 0.016), and DHCA time (26 [25–28] vs. 22 [19–25] min, *p* < 0.001) compared to the LSA Non-Occlusion Group. No significant differences were found in cardiopulmonary bypass time, time to extubation, postoperative ICU stay, length of hospital stay, or early (24-hour) markers of renal function (creatinine), perfusion (lactate), and pulmonary function (oxygenation index) (all *p* > 0.05).

**Table 2:** Intraoperative and early postoperative data.

Variable Names	LSA Non-Occlusion Group	LSA Occlusion Group	*p*
n	56	53	
Surgical duration	273 (248.75–295)	320 (295–350)	<0.001
Cardiopulmonary Bypass Time	159 (135.75–176.5)	161 (134–184)	0.141
Aortic cross-clamp time	104.5 (88.5–122)	126 (101–130)	0.016
DHCA time	22 (19–25)	26 (25–28)	<0.001
Time for tracheal intubation removal	23.5 (13.75–134.75)	14 (10–96)	0.143
Postoperative ward transfer time	46 (34–89.25)	34 (34–94)	0.157
Length of postoperative hospital stay	13.5 (10–20)	12 (9–16)	0.227
Creatinine Level within 24 h	96 (64.75–124.25)	101 (67–190)	0.725
Serum Lactate Level at 24 h	2.9 (1.475–6.425)	1.8 (1.4–6.4)	0.487
Oxygenation index within 24 h	198 (129.5–307.5)	200 (142–299)	0.811

### 3.3 Postoperative complications and outcomes

The incidence of postoperative complications and 30-day outcomes is detailed in Table 3. Categorical variables were analyzed using the Chi-square test or Fisher's exact test, as appropriate. The most notable finding was a significant difference in the rate of perioperative cerebral infarction, which was higher in the LSA Occlusion Group (22.64% vs. 7.14%, *p* = 0.044). Although the 30-day mortality rate was numerically higher in the LSA Occlusion Group (20.75% vs. 10.71%), this difference did not reach statistical significance (*p* = 0.238). Similarly, there were no significant differences between the two groups in the rates of multiple organ dysfunction syndrome (MODS), tracheostomy, hemodialysis, postoperative acute kidney injury, arteriovenous thrombosis, incision infection, perioperative myocardial infarction, postoperative hoarseness, secondary thoracotomy, postoperative atrial fibrillation, or pleural effusion (all *p* > 0.05).

**Table 3:** Postoperative complications and outcomes.

Variable Names	Level	LSA Non-Occlusion Group	LSA Occlusion Group	*p*
n		56	53	
30-Day Mortality (%)	No	50 (89.29)	42 (79.25)	0.238
	Yes	6 (10.71)	11 (20.75)	
MODS (%)	No	55 (98.21)	52 (98.11)	1.000
	Yes	1 (1.79)	1 (1.89)	
Tracheostomy (%)	No	46 (82.14)	45 (84.91)	0.896
	Yes	10 (17.86)	8 (15.09)	
Hemodialysis (%)	No	49 (87.50)	41 (77.36)	0.253
	Yes	7 (12.50)	12 (22.64)	
Postoperative Acute Kidney Injury (%)	No	44 (78.57)	39 (73.58)	0.700
	Yes	12 (21.43)	14 (26.42)	
Postoperative arteriovenous thrombosis (%)	No	52 (92.86)	51 (96.23)	0.726
	Yes	4 (7.14)	2 (3.77)	
Incision infection (%)	No	54 (96.43)	51 (96.23)	1.000
	Yes	2 (3.57)	2 (3.77)	
Perioperative myocardial infarction (%)	No	55 (98.21)	51 (96.23)	0.961
	Yes	1 (1.79)	2 (3.77)	
Hoarseness after surgery (%)	No	54 (96.43)	51 (96.23)	1.000
	Yes	2 (3.57)	2 (3.77)	
Secondary thoracotomy (%)	No	54 (96.43)	47 (88.68)	0.237
	Yes	2 (3.57)	6 (11.32)	
Postoperative atrial fibrillation (%)	No	54 (96.43)	53 (100.00)	0.500
	Yes	2 (3.57)	0 (0.00)	
Postoperative pleural effusion (%)	No	51 (91.07)	49 (92.45)	1.000
	Yes	5 (8.93)	4 (7.55)	
Perioperative cerebral infarction (%)	No	52 (92.86)	41 (77.36)	0.044
	Yes	4 (7.14)	12 (22.64)	

### 3.4 Sensitivity analysis

To evaluate the robustness of the primary outcome (perioperative cerebral infarction) and assess the fragility of the reported statistical significance (*P* = 0.044), we performed a sensitivity analysis by artificially changing the outcome status of a single patient. We tested two extreme scenarios to determine the minimum number of event changes required to alter the statistical conclusion.

#### 3.4.1 Key finding

The statistical significance of the observed difference in perioperative cerebral infarction rates depends on a single outcome event. Adding one cerebral infarction to the LSA Non-Occlusion Group (from 4 to 5 events) would render the difference not statistically significant (*P* = 0.105). Similarly, subtracting one event from the LSA Occlusion Group (from 12 to 11 events) would also eliminate the statistical significance (*P* = 0.129). The results of this sensitivity analysis are presented in Table 4.

**Table 4:** Sensitivity analysis of perioperative cerebral infarction.

Scenario	Group	No Cerebral Infarction	Cerebral Infarction	Rate of Cerebral Infarction	*P*-value
Original Data	LSA Non-Occlusion	52	4	7.14%	0.044
	LSA Occlusion	41	12	22.64%	
Scenario 1: +1 Event in Non-Occlusion Group	LSA Non-Occlusion	51	5	8.93%	0.105
	LSA Occlusion	41	12	22.64%	
Scenario 2: −1 Event in Occlusion Group	LSA Non-Occlusion	52	4	7.14%	0.129
	LSA Occlusion	42	11	20.75%	

This analysis confirms that the statistically significant difference in perioperative cerebral infarction rates between the two groups is highly event-driven and fragile. The result should be interpreted as an exploratory finding that requires validation in larger, adequately powered studies.

### 3.6 Multivariable analysis

To identify independent risk factors for perioperative cerebral infarction, a multivariable logistic regression analysis was performed. The model included LSA occlusion strategy (LSA Occlusion Group vs. LSA Non-Occlusion Group) and deep hypothermic circulatory arrest (DHCA) time, based on clinical relevance and the limited number of events (*n* = 16). The results are presented in Table 5.

**Table 5:** Multivariable logistic regression analysis for perioperative cerebral infarction.

Variable	Univariable OR (95% CI)	Univariable *P*-value	Multivariable OR (95% CI)	Multivariable *P*-value
LSA Occlusion (vs. Non-Occlusion)	3.73 (1.12–12.40)	0.032	4.36 (1.24–15.30)	0.020
DHCA time(per 1 min increase)	1.08 (0.99–1.18)	0.072	1.05 (0.96–1.16)	0.269

After adjustment, LSA occlusion remained a significant independent risk factor for perioperative cerebral infarction (OR = 4.36, 95% CI 1.24–15.30, *P* = 0.020). In contrast, DHCA time was not independently associated with cerebral infarction in the multivariable model (OR = 1.05, 95% CI 0.96–1.16, *P* = 0.269). This finding suggests that the increased stroke risk is primarily attributable to the LSA clamping strategy itself, rather than simply to the longer DHCA time associated with the more extensive dissection.

The multivariable model was adjusted for LSA occlusion strategy and deep hypothermic circulatory arrest (DHCA) time, based on clinical relevance and the limited number of events (*n* = 16).

## 4 Discussion

Acute type A aortic dissection (ATAAD) represents a catastrophic cardiovascular emergency characterized by rapid progression and exorbitant mortality rates, necessitating immediate surgical intervention as the sole life-saving measure (1). While deep hypothermic circulatory arrest (DHCA) combined with hemiarch replacement has become the standard operative strategy, optimizing cerebral protection and minimizing ischemic insults remain paramount challenges (13). The management of supra-aortic branch vessels during arch reconstruction continues to generate considerable debate, particularly regarding the necessity of manipulating the left subclavian artery alongside the innominate and left common carotid arteries (13). Refining these technical nuances is critical, as even marginal reductions in circulatory arrest duration can significantly mitigate the risk of irreversible neurological injury and systemic organ dysfunction (14).

This retrospective cohort study specifically evaluated the clinical impact of omitting left subclavian artery clamping during hemiarch replacement for ATAAD, contrasting this simplified approach with the conventional three-vessel occlusion technique. By isolating this specific procedural variable within a homogeneous surgical cohort, we aimed to elucidate its influence on operative efficiency and early postoperative outcomes without the confounding effects of varying arch extents (6). Our analysis revealed that preserving the left subclavian artery significantly abbreviated total operative time and, crucially, reduced the duration of deep hypothermic circulatory arrest. These findings underscore the importance of surgical expediency in mitigating the profound physiological stress associated with prolonged circulatory arrest (15).

The observed reduction in DHCA duration achieved by omitting LSA isolation represents a critical modification in surgical workflow, directly addressing the pathophysiological cascade triggered by prolonged systemic cooling and rewarming. Extended DHCA periods are mechanistically linked to exacerbated global ischemia-reperfusion injury, which precipitates a profound systemic inflammatory response syndrome characterized by endothelial glycocalyx shedding, leukocyte activation, and subsequent multi-organ dysfunction (16). Furthermore, the correlation between extended cardiopulmonary bypass and arrest times with postoperative hypoxemia suggests that minimizing these intervals preserves pulmonary vascular integrity and reduces extravascular lung water accumulation (17, 18). While some literature advocates for extensive arch replacement to prevent distal reoperation, such aggressive approaches invariably prolong arrest times, potentially offsetting long-term benefits with immediate perioperative risks in the acute setting (2). Consequently, the simplified two-vessel strategy effectively mitigates the dose-dependent toxicity of deep hypothermia, prioritizing the preservation of cellular homeostasis over anatomical comprehensiveness during the acute phase.

The observed association between LSA occlusion and increased perioperative stroke may be mediated by multiple mechanisms. First, the dissection and clamping of the LSA may dislodge atheromatous debris or fresh thrombus from the LSA ostium, causing embolic stroke to the posterior circulation (vertebrobasilar territory). Second, during selective antegrade cerebral perfusion (SACP), the LSA, when left open, can serve as an additional inflow pathway to the posterior circulation via the left vertebral artery. In patients with an incomplete Circle of Willis, the loss of this collateral route during LSA occlusion may lead to hypoperfusion of the posterior brain regions, particularly during prolonged DHCA. Third, the longer DHCA time and cross-clamp time in the LSA occlusion group are not simply a cause of ischemic injury, but rather a marker of a more complex and technically demanding procedure. The additional manipulation of the arch vessels may increase the risk of embolization and hemodynamic instability. Therefore, even though the absolute difference in DHCA time was modest (4 min), the cumulative effect of surgical trauma and altered perfusion dynamics may underlie the higher stroke rate. Future studies incorporating intraoperative cerebral oximetry and postoperative diffusion-weighted MRI could help elucidate these mechanisms.

Regarding neurological outcomes, the significant disparity in perioperative cerebral infarction rates between the three-vessel and two-vessel groups underscores the delicate balance between embolic protection and perfusion adequacy during arch manipulation. The unadjusted increase in stroke risk with extensive vessel handling likely stems from increased opportunities for atheroembolization or air microemboli release during the complex dissection and clamping of the LSA (19). This observation contrasts with findings suggesting that total arch replacement does not inherently elevate stroke risk compared to hemiarch repair, implying that the specific technical complexity of manipulating the third vessel, rather than the extent of replacement itself, may be the contributing factor in our cohort (8). The mechanism of injury here appears less related to prolonged ischemia, given the protective effects of hypothermia, and more attributable to mechanical disruption of the fragile dissected intima or plaque debris during the additional clamping maneuvers required for the third vessel.

Prolonged operative exposure facilitates a vicious cycle of coagulopathy, hemodilution, and complement activation, which collectively impair tissue oxygen delivery and exacerbate reperfusion injury upon rewarming (20). This temporal dependency is consistent with reports demonstrating that frozen elephant trunk techniques, while offering distal aortic protection, incur higher transfusion requirements and longer arrest times that may negate survival advantages in the absence of specific indications like malperfusion (20). Interestingly, while extended arch replacement has been associated with lower rates of late reoperation due to distal anastomotic new-entry tears, our data suggest that in the acute setting, the immediate mortality and morbidity driven by time-related organ failure outweigh the potential long-term benefits of a more extensive initial repair (11).

### 4.1 Limitations

This study has several limitations that warrant consideration. First, the retrospective, single-center design inherently introduces selection and information biases. The non-randomized allocation of surgical strategies, although with balanced baseline characteristics, cannot fully eliminate residual confounding from unmeasured factors such as nuanced variations in surgical technique, anesthesia protocols, or intraoperative decision-making. Second, the sample size, while adequate for detecting differences in primary time-based endpoints, may be underpowered to definitively assess the impact on relatively low-incidence but critical clinical outcomes, such as mortality or specific neurological complications. This limitation constrains the generalizability of our findings regarding safety endpoints and necessitates cautious interpretation of the non-significant associations in the multivariate model for certain complications. Third, the sensitivity analysis demonstrated that the observed difference in cerebral infarction rates is fragile, dependent on a single outcome event. Therefore, the stroke risk difference should be interpreted as an exploratory finding that requires validation in larger, adequately powered studies. Fourth, we did not systematically assess the anatomy of the Circle of Willis or the status of the vertebral arteries, which may influence the risk of posterior circulation stroke. Finally, long-term follow-up data on neurological outcomes and distal aortic remodeling were not available.

In conclusion, this analysis demonstrates that a simplified surgical strategy omitting LSA manipulation during hemiarch replacement for ATAAD significantly reduces core operative times, particularly the critical DHCA interval. Multivariable analysis identified LSA occlusion as an independent risk factor for perioperative cerebral infarction. However, a sensitivity analysis revealed that the statistical significance of this stroke difference is highly fragile, depending on a single outcome event. Therefore, while the LSA-sparing strategy clearly improves operative efficiency, the observed difference in stroke risk should be interpreted as an exploratory finding requiring validation in larger, adequately powered prospective studies. Future studies should incorporate standardized long-term follow-up protocols (e.g., at 1, 3, and 5 years) to evaluate the durability of the two surgical strategies, including all-cause mortality, aortic reintervention, neurologic events, and quality of life. The use of imaging-based assessment of false lumen thrombosis and aortic remodeling would also provide crucial insights into the biological impact of LSA management.

## Data Availability

The raw data supporting the conclusions of this article will be made available by the authors, without undue reservation.
