# Correction: Impact of left subclavian artery management on surgical efficiency and perioperative cerebral infarction in type A aortic dissection under deep hypothermic circulatory arrest: a single-center retrospective cohort study

**DOI:** 10.3389/fcvm.2026.2005914

**Published:** 2026-10-08

**Authors:** Yun Lu, Haisu Xu, Yang Zhang, Jun Wei, Fangyuan Chen

**Affiliations:** 1 Department of Cardiology, The Second Affiliated Hospital of Xuzhou Medical University, Xuzhou, China; 2 Department of Cardiac Surgery, Xuzhou Central Hospital, Southeast University, Xuzhou, China; 3 Department of Cardiothoracic Surgery, Affiliated Hospital of Xuzhou Medical University, Xuzhou, China

**Keywords:** aortic dissection, cerebral infarction, deep hypothermic circulatory arrest (DHCA), hemiarch replacement, left subclavian artery (LSA)

In the published article, the following funding information was erroneously omitted: the project “One-stop Coronary CCTA and AI-assisted Precise Grading and Prediction of Coronary Heart Disease” (Grant No. GSPJS202518) and the Key Project of Jiangsu Provincial Health Commission (Grant No. K2024014), both awarded to Jun Wei.

The published Funding statement read:

“The author(s) declared that financial support was not received for this work and/or its publication.”

The corrected Funding statement reads:

The author(s) declared that financial support was received for this work and/or its publication. This work was supported by the Affiliated Hospital of Xuzhou Medical University through the project “One-stop Coronary CCTA and AI-assisted Precise Grading and Prediction of Coronary Heart Disease” (Grant No. GSPJS202518), and by the Key Project of Jiangsu Provincial Health Commission (Grant No. K2024014), both awarded to Jun Wei.

The original version of this article has been updated.

